# Risk prediction model construction for post myocardial infarction heart failure by blood immune B cells

**DOI:** 10.3389/fimmu.2023.1163350

**Published:** 2023-05-23

**Authors:** HouRong Sun, XiangJin Kong, KaiMing Wei, Jie Hao, Yue Xi, LingWei Meng, GuanNan Li, Xin Lv, Xin Zou, XingHua Gu

**Affiliations:** ^1^ Qilu Hospital, Cheeloo College of Medicine, Shandong University, Jinan, Shandong, China; ^2^ Department of Cardiovascular Surgery, Qilu Hospital of Shandong University, Jinan, Shandong, China; ^3^ Institute of Clinical Science, Zhongshan Hospital, Fudan University, Shanghai, China; ^4^ Department of Reproductive Medicine, Central Hospital Affiliated to Shandong First Medical University, Jinan, China; ^5^ Jinshan Hospital Center for Tumor Diagnosis & Therapy, Jinshan Hospital, Fudan University, Shanghai, China

**Keywords:** myocardial infarction (MI), heart failure (HF), B cells, risk prediction model, single cell RNA-seq

## Abstract

**Background:**

Myocardial infarction (MI) is a common cardiac condition with a high incidence of morbidity and mortality. Despite extensive medical treatment for MI, the development and outcomes of post-MI heart failure (HF) continue to be major factors contributing to poor post-MI prognosis. Currently, there are few predictors of post-MI heart failure.

**Methods:**

In this study, we re-examined single-cell RNA sequencing and bulk RNA sequencing datasets derived from the peripheral blood samples of patients with myocardial infarction, including patients who developed heart failure and those who did not develop heart failure after myocardial infarction. Using marker genes of the relevant cell subtypes, a signature was generated and validated using relevant bulk datasets and human blood samples.

**Results:**

We identified a subtype of immune-activated B cells that distinguished post-MI HF patients from non-HF patients. Polymerase chain reaction was used to confirm these findings in independent cohorts. By combining the specific marker genes of B cell subtypes, we developed a prediction model of 13 markers that can predict the risk of HF in patients after myocardial infarction, providing new ideas and tools for clinical diagnosis and treatment.

**Conclusion:**

Sub-cluster B cells may play a significant role in post-MI HF. We found that the *STING1, HSPB1, CCL5, ACTN1*, and *ITGB2* genes in patients with post-MI HF showed the same trend of increase as those without post-MI HF.

## 1 Introduction

Myocardial infarction (MI), the result of the rupture or erosion of a vulnerable, lipid-filled, chronic atherosclerotic coronary plaque leading to the acute disruption of myocardial blood flow and ischemic myocardial necrosis, remains a common cardiac emergency with high morbidity and mortality worldwide (1, 2). Despite significant advances in the treatment of coronary artery disease and acute MI over the past two decades, heart failure (HF) due to MI and the development of post-MI HF remain among the most common causes of cardiovascular death (3, 4). Acute inflammation caused by plaque rupture in acute coronary syndrome and MI triggers a subsequent response that may lead to long-term cardiac injury. There are differences in the degree of inflammatory recovery after coronary interventions for stenting or thrombolytic reperfusion, which have prognostic value in the development of ischemic HF (5).

Numerous studies have analyzed circulating biomarkers in the blood of post-MI patients, which reflect different inflammatory traits, coagulation activity, endothelial dysfunction, atherogenesis, myocardial dysfunction and injury, apoptosis, renal function, glucose, and lipid metabolism, to explore factors that can predict poor outcomes after MI (6–8). Although several genes, such as natriuretic peptides, galactose lectin-3, and soluble tumorigenic suppressor-2 have been reported as biomarkers for HF, their reliability remains controversial (9, 10). These studies indicate that elevated levels of circulating pro-inflammatory biomarkers in patients with HF correlate with disease severity and prognosis. Experimental studies have shown that the activation of cardiac immune response mechanisms triggers adverse cardiac remodeling and leads to left ventricular dysfunction (11). Therefore, the development of novel powerful immune-related biomarkers with predictive potential to screen for the incidence of ischemic pretreatment and MI and the development of HF after MI remains a key goal for scientific advances in cardiovascular disease.

Inflammatory cells in the peripheral blood of MI patients may play a significant role (12). After MI, myeloid cells and T lymphocytes in the blood display distinct disparities in abundance (13, 14). Following MI, classical and non-classical blood monocytes comprise the majority of inflammatory cell types, with NK and B cells being crucial in the activation of inflammatory cells and the promotion of chemokine synthesis in plaque rupture (15). Notably, this distinction between the immune cells of patients who experienced HF after MI and those who did not was significant (16). Currently, there are no immune-inflammatory biomarkers that can be used to clinically predict the risk of post-MI HF.

In recent years, researchers have advanced our understanding of microenvironmental alterations in tissues with different HF etiologies through single cell RNA sequencing (scRNA-seq) analysis and other experimental approaches (17–19). However, most studies have focused on the analysis of altered cell types and pathological pathways within cardiac tissues, and few have explored the prognosis of cardiac disease through alterations in immune cells in the blood. In this study, we reanalyzed published single-cell and bulk RNA-seq data from HF patients with post-MI HF to construct a prediction model. We found that a group of immune-activated B cells in blood may play an important role in disease progression. Based on these findings, we developed an effective post-MI HF risk-prediction signature using 13 genes. Through further analysis, the five genes with the highest diagnostic value: *STING1, HSPB1, CCL5, ACTN1*, and *ITGB2* were identified and further validated by polymerase chain reaction. These findings extend our understanding of the factors associated with the development of HF after MI and provide a potentially reliable predictive model for the development of HF after MI.

## 2 Materials and methods

### 2.1 Collection of bulk RNA-seq and scRNA-seq datasets

To investigate immunocyte subtypes and characteristics that are highly predictive of HF after MI, we obtained and re-examined a previously published heart failure after MI scRNA-seq dataset (17), and validated the results in multiple MI bulk RNA-seq datasets, and an HF after MI bulk RNA-seq (12, 16). The single-cell dataset (17) consisted of blood samples from patients with HF after MI and healthy subjects for single-cell sequencing analysis. The processing, clustering, and cell-type determination procedures of the scRNA-seq datasets, as well as the extensive clinicopathological data for every patient in the dataset, were thoroughly discussed in their original studies.

We used two bulk MI datasets, one comprising blood from 14 MI patients and 10 normal subjects, and another dataset containing blood from 17 MI patients and seven normal subjects. Samples containing post-MI HF and post-MI non-HF in GSE59867 (16) were then used for further screening, which included eight patients with post-MI HF and nine post-MI non-HF patients. Blood samples from each patient in this dataset were selected for analysis at four time points: admission, discharge, 1 month post-MI and 6 months post-MI. The Hallmark, Kyoto Encyclopedia of Genes and Genomes (KEGG), Gene Ontology Biological Process (GOBP), and Reactome gene sets were retrieved for Gene Set Enrichment Analysis from the Molecular Signatures Database (MSigDB) (http://www.gsea-msigdb.org/gsea/index.jsp), which was the database used in our investigation.

### 2.2 Study design

We extracted 6 cell types from the GSE145154 (17) scRNA-seq dataset. Due to the small number of hematopoietic stem cells, we targeted only the remaining five immune cells in subsequent analyses. We used the scCODE (20) v1.0.0.0 R package provided by Zou et al. to determine the differentially expressed (DE) genes between normal and post-MI HF patients for each of the aforementioned five cell types. The reliability of the single-cell DE analysis was improved by scCODE’s ability to examine the chosen DE genes using a range of testing methods. Therefore, we obtained a list of five DE genes. We used the Investigate Gene Sets tool (http://www.gsea-msigdb.org/gsea/msigdb/annotate.jsp) to compute the enriched gene sets between our gene lists and the gene sets in MSigDB. For each of the five DE gene lists identified by scCODE (20), we sorted them according to the absolute value of the logFC of each DE gene from largest to smallest and submitted the top 500 genes of each DE gene list to Investigate Gene Sets tool (all genes were submitted if less than 500). Each DE gene list can obtain several gene sets enriched in GOBP, Hallmark, KEGG, and Reactome using the default parameters of the tool (display the top 10 genes and false discovery rate (FDR) q-value less than 0.05). Using the Cancerclass (21) v1.34.0 R program, the prediction power of these enriched gene sets was evaluated. Using the same program, we evaluated the capacity of the following gene sets to predict post-MI outcomes of GSE59867 (16) (n.patients: HF = 8, non-HF = 9, total of 32 HF samples and 36 non-HF samples). The prediction sensitivity and specificity were evaluated in terms of the receiver operating characteristic (ROC) curve and matching area under the curve (AUC). The Cancerclass R package was used to create and validate the high-dimensional molecular data categorization tests. Feature selection and nearest centroid classification were performed sequentially. To obtain continuous prediction scores, multiple random validation methodology was used to verify the categorization results. The success of the classifier’s classification results was indicated by the p-value of the ROC curve, which was obtained using Welch’s t-test included in the Cancerclass R package. Each gene set was evaluated independently using a classifier.

### 2.3 Single-cell RNA sequencing data processing

We extracted and defined cell types from the post-MI HF single-cell dataset GSE145154 (17). We used the Seurat v4.0.4 R package (22) to identify cell types and subtypes. The extracted dataset was first combined across the samples. Prior to this, the expression of each gene was scaled to a scale factor of 10,000, converted logarithmically (Seurat’s default method), and normalized to the total expression in the associated cell. FindVariableFeatures was used to identify the top 2000 variable features, which were then used in further studies. The mutual nearest-neighbor approach was then used to correct for batch effects (23), and the percentage of mitochondrial transcripts was regressed using ScaleData. The integrated assay was only used for dimension reduction and clustering, and raw log-normalized expression data were used for all DE and gene-level analyses. Principal component analysis was performed on the integrated assay using RunPCA. Cell clustering was performed using the first 20 main components, with a resolution parameter of 0.5. Finally, visualization was performed in two-dimensional space through unified manifold approximation and projection (Dims = 1:20).

### 2.4 Marker gene analysis

Marker gene analysis was performed for all clusters using FindAllMarkers included in the Seurat package (22), with the parameters min.pct = 0.1 and logfc.threshold = 0.25. Genes with p.adjust < 0.05 were selected as cluster-specific marker genes.

### 2.5 Gene enrichment and gene set variation analysis

We used the GSVA method with default settings to assign specific gene signature activity scores to individual cells or samples using the GSVA (v1.38.2) R package (24). Using FindMarkers built into the Seurat package (22), we compared all gene expression fold changes in disease and normal C4 subclusters of B cells to obtain a list of DE genes for gene enrichment analysis by setting the parameter logfc.threshold = -Inf, min.pct = -Inf, min.diff.pct = -Inf. Gene enrichment was performed on DE gene lists based on pre-downloaded Hallmark, KEGG, GOBP, and Reactome gene sets using the default parameters of the clusterProfiler v3.18.1 R package. This R package can also be used to examine whether a particular gene set is enriched at the top or bottom of a preordered gene list. The Benjamini-Hochberg method was used to compare two sets, and gene sets with FDR-adjusted p-values < 0.05 were deemed significantly enriched in one set.

### 2.6 Development of a gene signature associated with HF after MI

Using the Cancerclass v1.34.0 R package, we created gene signatures based on the cluster-specific genes of the C4 of B cell subtype. Specifically, using Cancerclass, the p-values of the ROC curves for the enriched GOBP gene sets in B cells were generated. They were adjusted for FDR using the Benjamini-Hochberg method. We combined all gene sets with p.adjust < 0.05, to create a total of 115 genes (the selected gene signature is referred to as HF.Sig in this study). The cyclic algorithm is shown in **Supplementary Figure 2**. In each cycle, 114 gene combinations were selected randomly from the signature. Cancerclass was then used to estimate the AUC in the GSE59867 (16) cohort and examine the prediction accuracy of each of these combinations. Of these 115 combinations, gene combinations with the highest AUC (genelist_maxAUC_) were retained and applied to the following cycle. This cycle was repeated until no more than three distinct gene combinations remained. Finally, the highest AUC values for gene panels in all loops were selected. A combination of appropriate gene numbers was selected as the new HF.Sig for subsequent analyses.

### 2.7 Heatmap of DE genes and protein-protein interactions

Generalized linear model approach of Limma v3.46.0 (25). package was used to calculate the fold-changes in gene expression between post-MI HF and non-HF patients. To plot the protein interaction nodes in accordance with the protein-protein interaction network map, we first took the overlapping genes from MI-related cell cluster markers from a single-cell dataset and differential genes from the GSE59867 (16) bulk dataset, uploaded them to the STRING database website (https://cn.string-db.org), set a medium confidence interval (CI) (medium confidence = 0.4), exported the tab-separated values files, and then used the circlize package for drawing.

### 2.8 Real-time polymerase chain reaction analysis

Quantitative real-time polymerase chain reaction was used to analyze the relative expression of target genes. Following the manufacturer’s instructions, RNA was isolated from human blood mononuclear cells using the RNeasy Fibrous Tissue Mini Kit (QIAGEN, Hilden, Germany), and complementary DNA was synthesized using the High-Capacity complementary DNA Reverse Transcription Kit (Thermo Fisher Scientific, Waltham, MA). One microliter of complementary DNA, two microliters of forward and reverse primers, ten microliters of Fast SYBR Green Master Mix (Thermo Fisher Scientific), and eight microliters of nuclease-free water were added to a 20-microliter reaction mixture, which was then run through a real-time polymerase chain reaction system (Applied Biosystems, Foster City, CA) to quantify target genes by fluorescence. The primers used in quantitative real-time polymerase chain reaction were as follows: ITGB2, F 5’-ATGCTGGGCCTGCGCCC-3’, R 5’-GATGGTGTCACACTCGCAGTA-3’. STING1, F:5’- ATGCCCCACTCCAGCCTG -3’, R:5’- TCAAGCTGCCCACAGTAACCT -3’. ACTN1, F:5’-ATGGACCATTATGATTCTCAGCAAA-3’, R:5’-TTAGAGGTCACTCTCGCCGTA -3’. CCL5, F:5’-ATGAAGGTCTCCGCGGCAG-3’, R:5’-TCAAGGAGCGGGTGGGGTA-3’. HSPB1, F:5’- AGGAGTGGTCGCAGTGGTTAGG-3’, R:5’- CAGGGGACAGGGAGGAGGAAAC-3’. GAPDH, F:5’-ATCCCATCACCATCTTCC-3’ and R:5’-GAGTCCTTCCACGATACCA-3’.

### 2.9 Statistical analysis

We assessed the performance of each classifier in predicting post-MI HF outcomes by plotting their ROC curves, calculating their AUCs, and estimating their sensitivity and specificity using the Cancerclass framework. The p-value from the ROC curve was used to rank the classifiers in terms of reliability. We calculated 95% CIs for sensitivity and specificity using Wilson’s method implemented in Cancerclass and used Welch’s t-test to determine significance. Except where otherwise stated, all p-values were adjusted using the Benjamini-Hochberg procedure, and a corrected p-value of less than 0.05 was considered statistically significant. Wilcoxon’s test was used to determine how the variables were grouped. Binomial 95% CIs were used for all reported CIs. R v3.5.3 was used for all statistical analyses that were performed using this study.

## 3 Results

### 3.1 B cells, NK cells and monocytes are associated with the myocardial infarction

**Figure 1:**
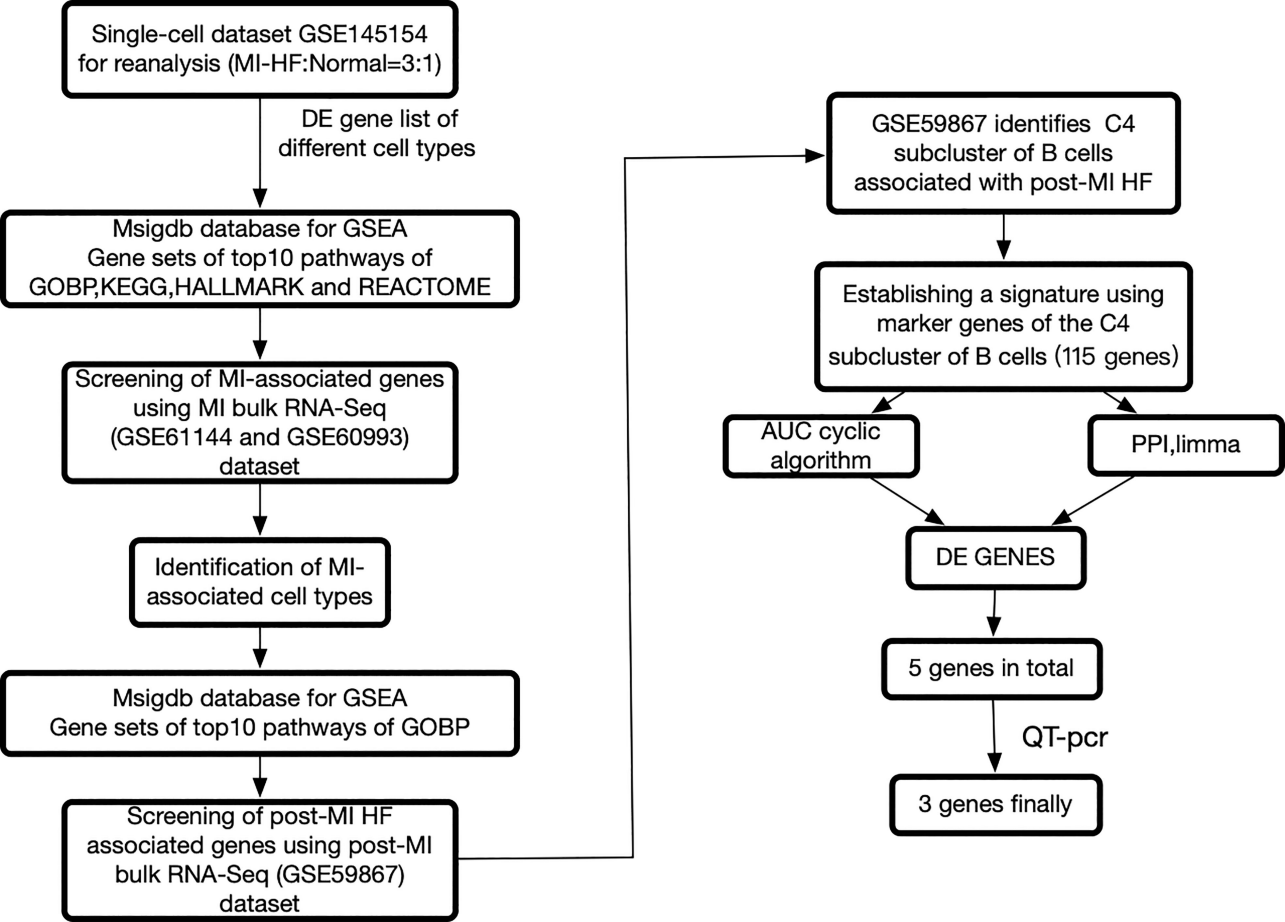
The framework of this research. Bulk RNA sequencing data can be found in GSE60993, GSE61144, and GSE59867, while single cell RNA sequencing data can be found in GSE145154. Myocardial infarction (MI), protein-protein interaction, differentially expressed genes, and heart failure all refer to gene sets that have been enriched in a specific way.

We re-analyzed publicly available heart failure scRNA-seq datasets from the blood of post-MI HF patients and healthy human controls. Multiple sets of MI bulk RNA-seq datasets and one set of post-MI HF bulk RNA-seq datasets were used for the validation. The workflow of this study is summarized in **Figure 1**. We carefully reprocessed the single-cell data based on the grouping of different individuals and whether it was a disease group and divided the cells into 27 sub-clusters (**Supplementary Figure 1**).

There were 6 cell types in the scRNA-seq dataset: CD4^+^ T cells, CD8^+^ T cells, NK cells, B cells, hematopoietic stem cells, and monocytes. **Supplementary Figure 2** shows the results of the visualization of the 6 immune cell types in two dimensions using uniform flow form approximation and projection. The locations of the characteristic genes for each cell type are shown in **Supplementary Figure 3**.

According to the workflow in **Figure 1**, we used scCODE on each individual cell type to identify the DE genes by comparing the post-MI HF and normal groups and obtained a total of 5 DE gene lists (due to the low number of hematopoietic stem cells, this group of cells was excluded from subsequent analyses). Only the DE genes that were upregulated in the disease group were used. From MSigDB, 5 DE genes from the disease group were found to be enriched in 200 gene sets. The calculation of these 200 gene sets is described in detail in the study design section.

**Figure 2:**
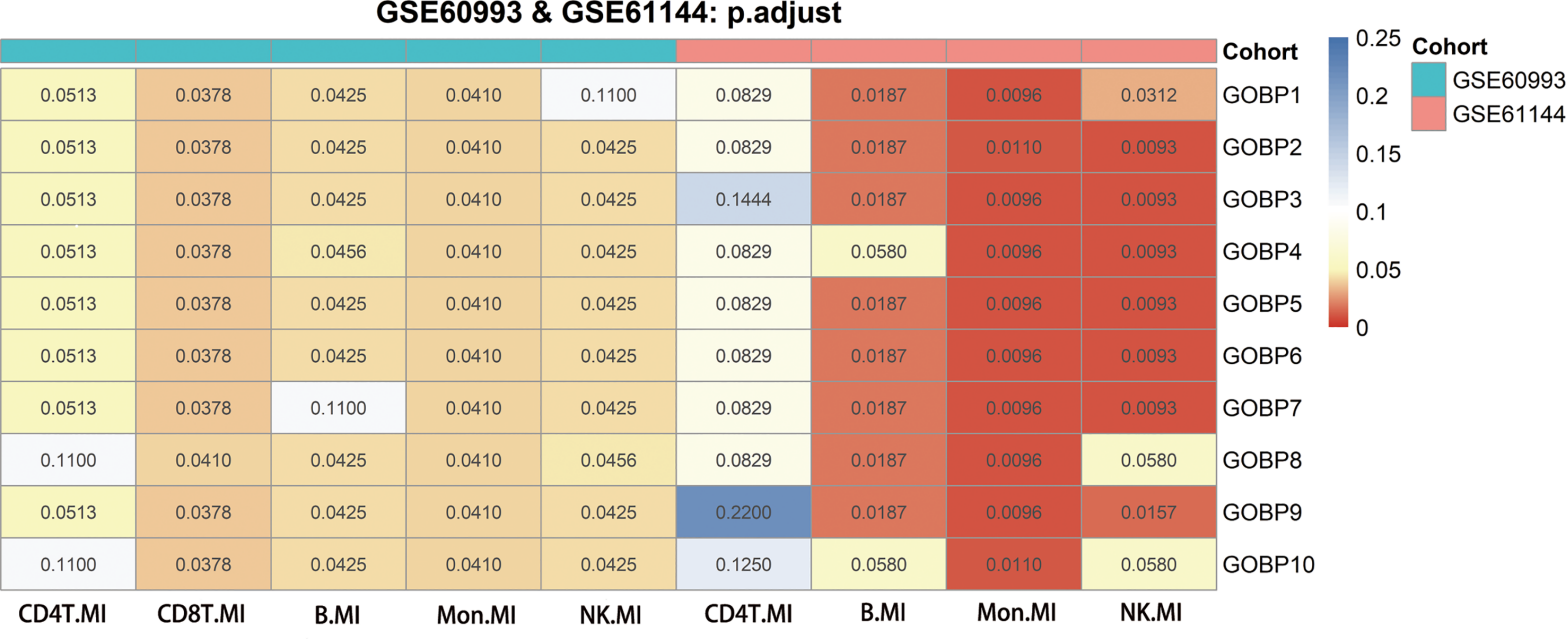
Genes from genomes enriched in nine significant differentially expressed genes using GSE60993, GSE61144 and their adjusted p-values for receiver operating characteristic curves. The Benjamini-Hochberg method was used to correct the p-values for the false discovery rate.

In comparison to Hallmark, KEGG, and Reactome, we discovered that the GOBP gene collection had a greater overall prediction capability (**Supplementary Figures 4A–D**, **Supplementary Figures 5A–D**). We explored the enriched GOBP gene sets (gene enrichment) with ROC p-values < 0.05 in each DE gene list to identify cell types with strong prediction potential. A DE gene list was considered significant if not less than half of the enriched gene sets had a ROC FDR < 0.05. We screened four relevant cells in the GSE60993 (12) bulk dataset: NK cells, CD8T cells, monocytes, and B cells. We screened three relevant cell types in the GSE61144 (12) bulk dataset: NK cells, monocytes, and B cells. By combining the results of the analysis of the two aforementioned datasets, we screened three cell types that may be associated with MI. These were NK cells, monocytes, and B cells (**Figure 2**).

### 3.2 B cells are associated with post-MI HF

**Figure 3:**
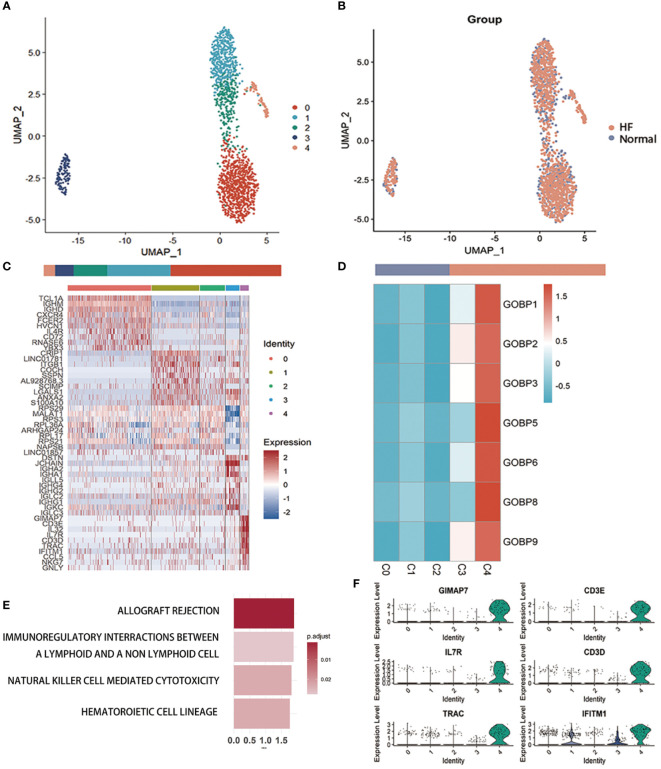
Examination of B cells in the MI dataset **(A)** Uniform Manifold Approximation and Projection plot of B cells in the dataset of GSE145154, with the sample divided into five subgroups. **(B)** Two subgroups were divided according to disease status. The bars show the proportion of cells grouped by cluster (left) and disease status (right). **(C)** Heatmap of scale-normalized expression of the top 10 specific marker genes for the B cell subclusters in GSE145154, identified by a two-sided Wilcoxon rank sum test with false discovery rate correction (p < 0.05). **(D)** MI prediction-related B subtype was identified by locating the effective predictive gene set expression *via* gene set variation analysis (GSVA). **(E)** Identification of MI prediction-associated B cell subclusters by GSVA to locate validly (receiver operating characteristic p.adjust < 0.05) predicted gene set expression. **(F)** violin plot of expression levels of marker genes specifically expressed in the B cell subclusters of GSE145154. A two-sided Wilcoxon test was used to determine the significance between the subclusters of interest and other subclusters. p < 0.0001.

We performed a subcluster analysis of the three cells mentioned above. First, B cells were divided into 5 subclusters using Seurat (22) (**Figure 3A**), and **Figure 3B** showed a scatter plot of the distribution of cells between the HF and normal groups. Following the discovery of cluster-specific marker genes using FindAllMarkers (Seurat) (22), **Figure 3C** presents an expression heatmap of the top 10 marker genes. Classical marker genes of B cells were highly expressed in all subclusters (**Supplementary Figure 6A**).

Seven gene sets of the DE genes of B cells with ROC p.adjust < 0.05. We used GSVA (24) to evaluate the expression of these gene sets in different B cell subclusters and discovered that they were substantially expressed in subcluster 4 (**Figure 3D**, **Supplementary Figure 6B**). Gene enrichment analysis of marker genes suggested that B cell-associated markers were enriched in graft rejection, immune interactions between lymphocytes and non-lymphoid cells, and NK cell cycle (**Figure 3E**). The violin plot lists the genes specifically expressed in the C4 cluster for further investigation of the B cell sub-cluster (**Figure 3F**, **Supplementary Figure 6C**). These genes, which are specifically expressed in the C4 sub-cluster of B cells, are associated with immune cell activation and development.

**Figure 4:**
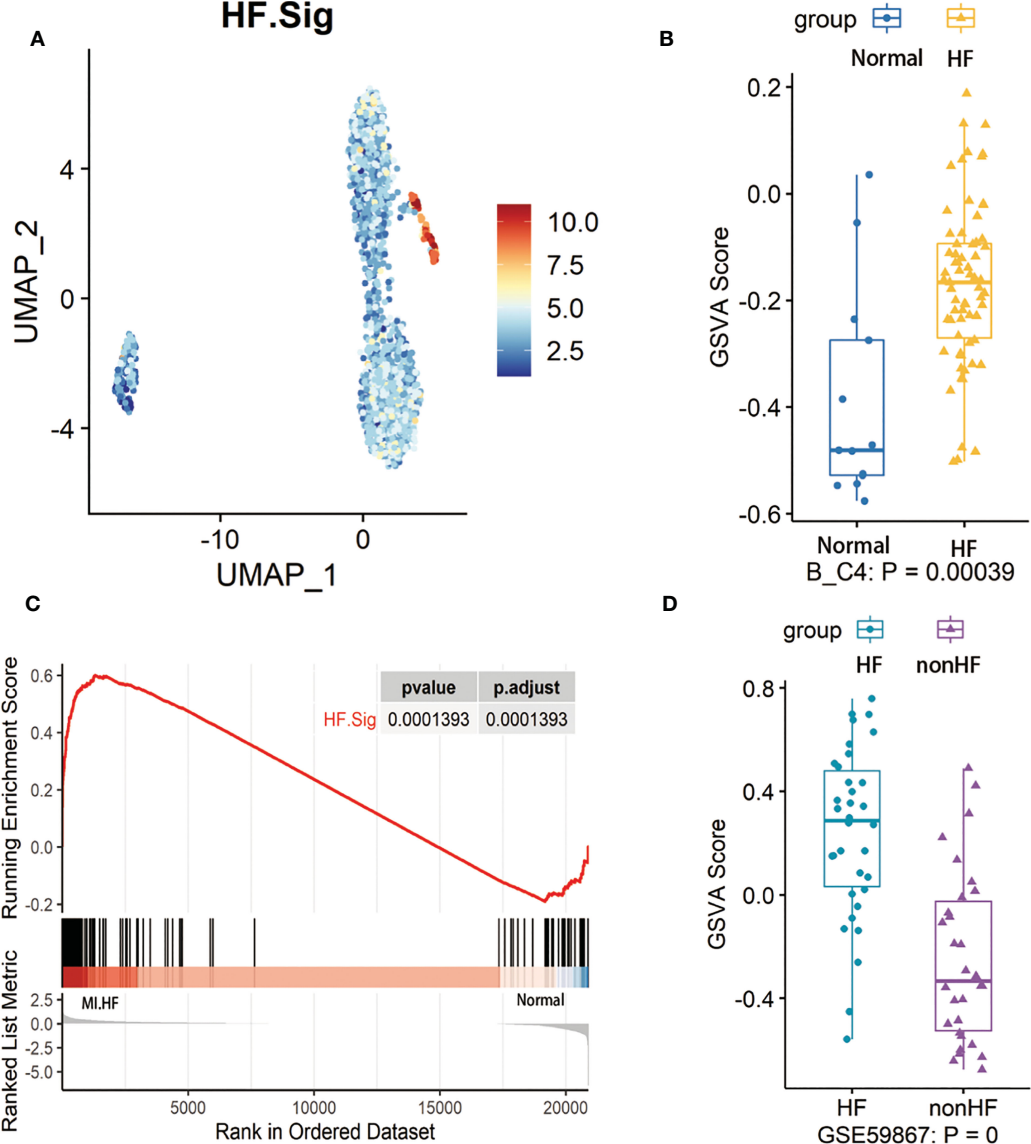
GSVA analysis of B cells and examination of HF.Sig **(A)** Feature map of the HF.Sig GSVA score showing that it can specifically characterize B cell C4 subcluster. **(B)** Boxplot found and validated significantly higher heart failure (HF) scores than normal scores in HF.Sig GSVA scores. Cohorts of B cells and **(C)** Gene set enrichment analysis found that HF.Sig was enriched in B_C4 was enriched in HF and normal. The p-value was false discovery rate-adjusted by the Benjamini-Hochberg method. **(D)** GSE59867 were verified by GSVA analysis to have significantly higher GSVA scores for HF than non-HF, center line, median, box limits, upper and lower quartiles. whiskers, 1.5 interquartile range. points beyond whiskers, outliers. A two-sided Wilcoxon test was used to determine significance.

**Figure 5:**
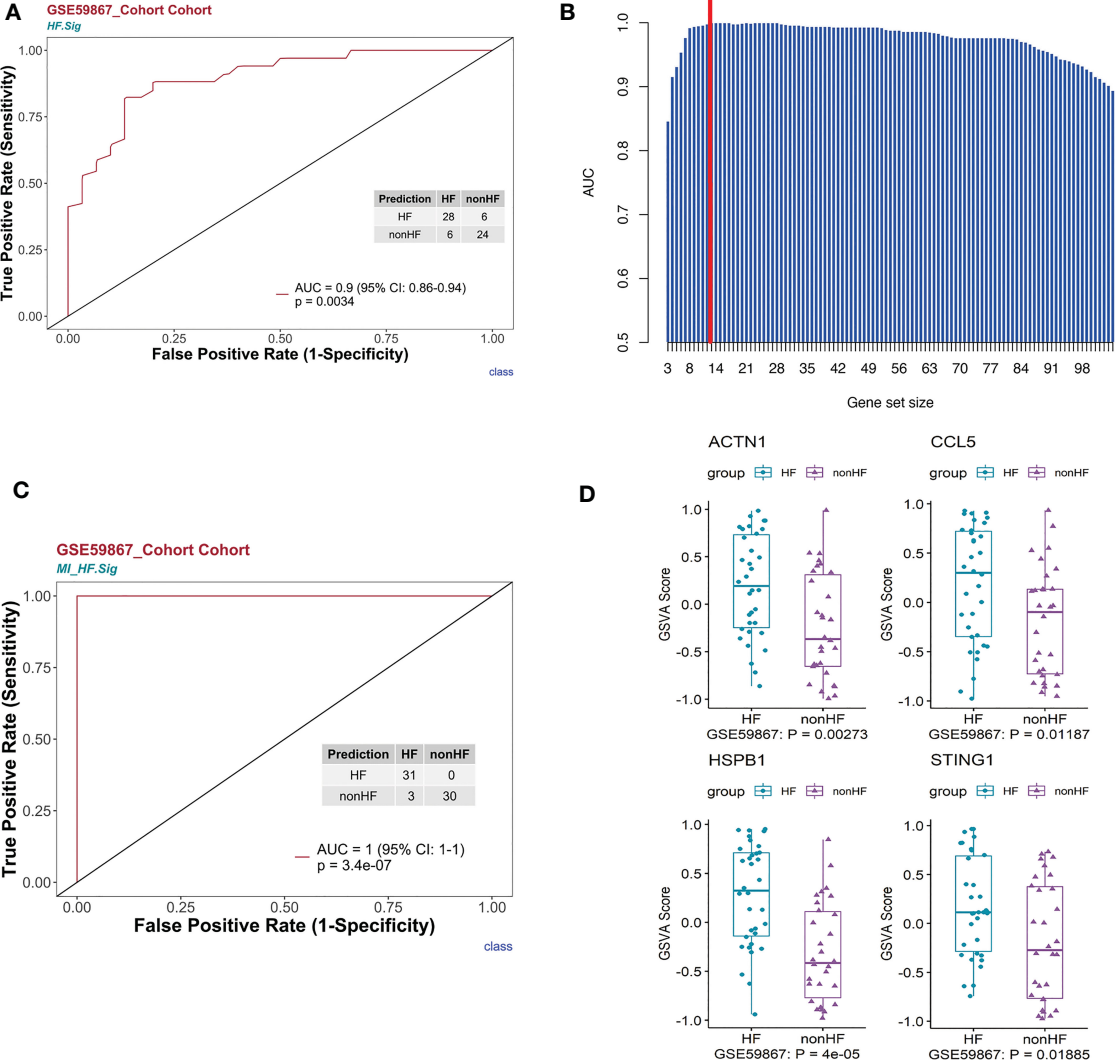
HF signatures are effective in predicting clinical outcomes in patients with MI. The bulk RNA-seq dataset GSE59867 was analyzed. **(A)** Univariate logistic regression model of HF.Sig in predicting MI outcomes. **(B)** Bar graph showing the area under the curve of gene combinations, maximum area under the curve per cycle (different gene-number combinations). Dashed line: 13-gene combination **(C)** The 13-gene combination had a significantly high predictive value for the outcome of whether HF occurred after MI in the GSE59867 cohort. **(D)** Evaluation of the above 13 genes using the GSVA score identified four genes that were found to be genes with increased expression for heart failure occurring after MI compared to HF not occurring after MI.

First, we performed gene enrichment analysis of marker genes in the C4 cluster of B cells and found that these marker genes in the C4 cluster were significantly predictive of the development of HF after MI (p < 0.05) (**Supplementary Figure 6D**). We also demonstrated that this gene signature (HF.Sig) is a good predictor of MI (**Supplementary Figure 6E**, **Supplementary Figure 6F**). The GSVA scores of HF.Sig were significantly higher than those of other subclusters of B cells (**Figure 4A**). **Figure 4B** shows the GSVA scores of HF.Sig in different B cell C4 subclusters in the disease and normal groups in different cells. These genes were enriched in patients (**Figure 4C**) and had significantly higher GSVA scores (**Figure 4D**). The gene sets were composed of B cell C4 cluster markers, and 115 genes were obtained from these gene sets. ROC curves for the 115 genes demonstrated a significant predictive power of the gene signature for the development of HF after MI, AUC = 0.9 (95% CI: 0.86–0.94. p = 0.0034) (**Figure 5A**).

### 3.3 Post-MI HF prediction model construction and validation

To construct a more effective predictive model for post-MI HF, we used a loop algorithm (**Supplementary Figure S7**) and calculated the AUCs for different numbers of genes. An AUC peak appeared for a combination of 13 genes (*CCL5, B2M, CD3D, CD6, CD79B, DGKZ, STING1, LYAR, KLRG1, PRKCA, HSPB1, ACTN1*, and *MYL12A*) (**Figure 5B**). HF.Sig consisting of 13 new genes could accurately distinguish between HF and non-HF in the GSE59867 (16) cohort, with an AUC of 1 (p = 7.2e-07, 95% CI:1-1) (**Figure 5C**).

Gene enrichment analysis was performed using the HFSig. Gene enrichment analysis showed that the first 20 pathways were mainly related to immune responses such as immune cell activation (26, 27) (**Supplement Figure 8A**). We performed ROC curve validation for the first 10 pathways and demonstrated good predictive performance for these pathways, with AUCs between 0.89 and 0.78 (**Supplementary Figure 8B**).

**Figure 6:**
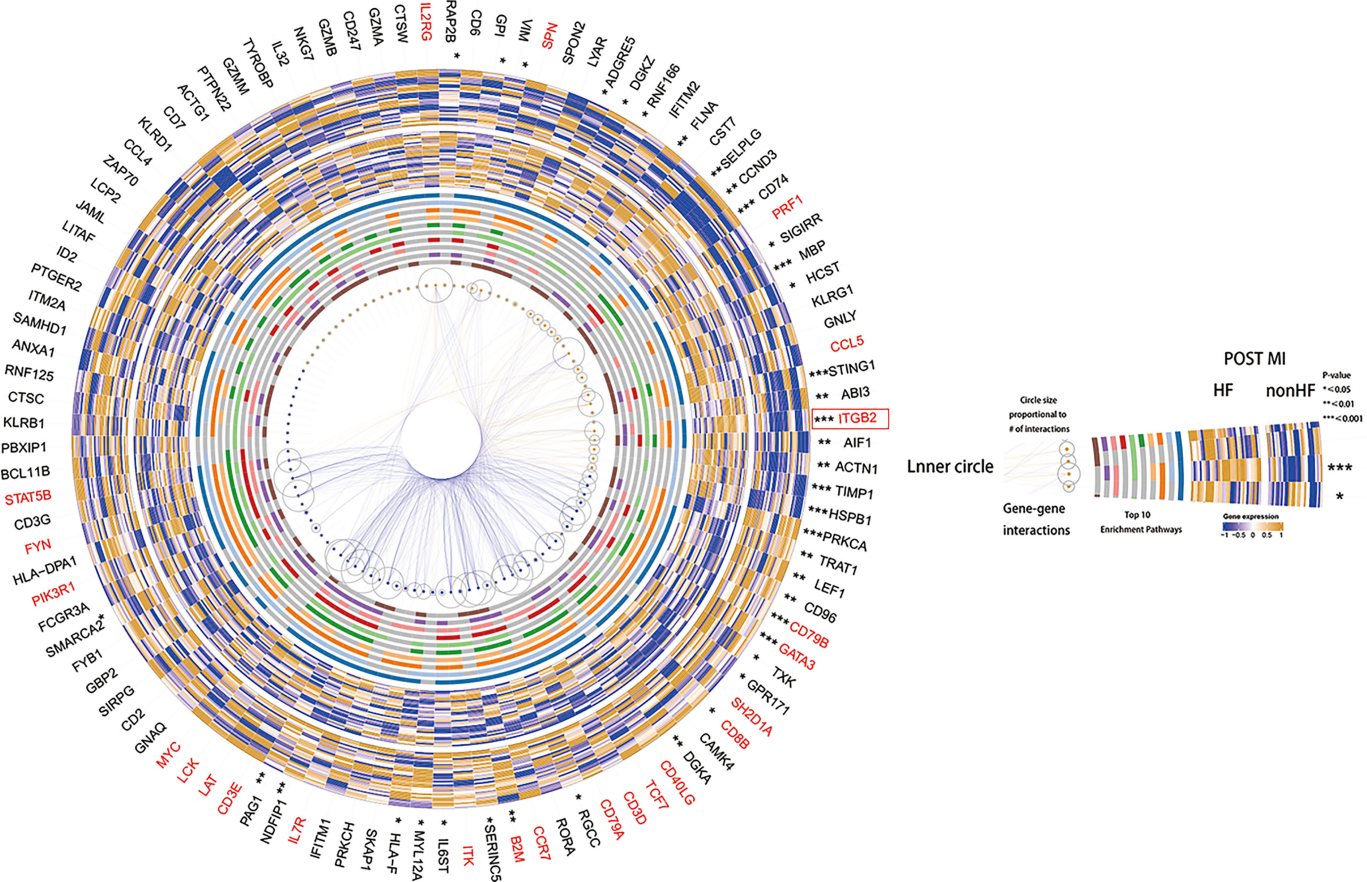
Heatmap made using the original HF.Sig of genes differentially expressed in cohort GSE59867, protein-protein interaction analysis and performance of the corresponding enriched Top10 pathway.

GSVA scoring of the above 13 gene signatures in GSE59867 (16) (n.patients: HF = 8, non-HF = 9, total of 32 HF samples, 36 non-HF samples) showed that eight of these genes were upregulated in HF relative to the non-HF sample for expression and found that four genes: *HSPB1, ACTN1, STING1*, and *CCL5*, were significant (**Figure 5D**, **Supplementary Figure 8C**). **Supplementary Figure 8D** shows the GSVA scores of four genes (*KLRG1, LYAR, CD6*, and *DGKZ*) in GSE59867 (16), which exhibited a similar trend. In addition, we considered the overlap between all DE genes from the dataset GSE59867 (16) and 115 genes from the B_C4 marker genes for the analysis. We obtained a total of 84 genes. We used these 84 genes for protein interaction network analysis using the STRING online tool. Among them, the upregulated gene with p < 0.05 and multiple linker proteins greater than 10 was *ITGB2*, which may play an important role in HF patients after MI (**Figure 6**).

Using the above algorithm, we demonstrated that the C4 subcluster of B cells could be used as a cell subcluster to predict the occurrence of HF after MI. However, monocyte and NK cell counts failed to accurately predict that HF will occur after MI. Monocytes were divided into five sub-clusters (**Supplementary Figure 9A**). However, monocytes were not able to localize to an exact sub-cluster, which can accurately predict MI (**Supplementary Figure 9D**). NK cells were divided into three sub-clusters (**Supplementary Figure 10A**). **Supplementary Figure 10B** shows a scatter plot of the distribution of cells between the disease and normal groups. **Supplementary Figure 10C** presents an expression heatmap of the top ten marker genes. We analyzed the expression of NK gene sets in the MI dataset using GSVA and found that they were highly expressed in NK C1 (**Supplementary Figure 10D**). The performance of five DE gene list-enriched gene sets was assessed for predicting post-MI HF in MI-related cell types. The C1 cell subclusters showed good predictive performance (**Supplementary Figure 11A**). However, these gene sets failed to accurately reflect enrichment in post-MI HF (p = 0.2933, p.adjust = 0.2933) (**Supplementary Figure 11B**). The feature plot of the HF.Sig GSVA score showed that it could not specifically describe the NK.Sig (**Supplementary Figure 11C**). We performed GSVA of the gene sets in C1 NK cells (**Supplementary Figure 11D**) (Wilcoxon rank-sum test p = 0.92707). The GSVA scores for these gene sets failed to meet the requirement for distinguishing between the two groups of samples.

### 3.4 Expression of *CCL5, STING, HSPB1, ACTN1* and *ITGB2* genes in blood samples from post-MI HF patients and post-MI non-HF patients

**Figure 7:**
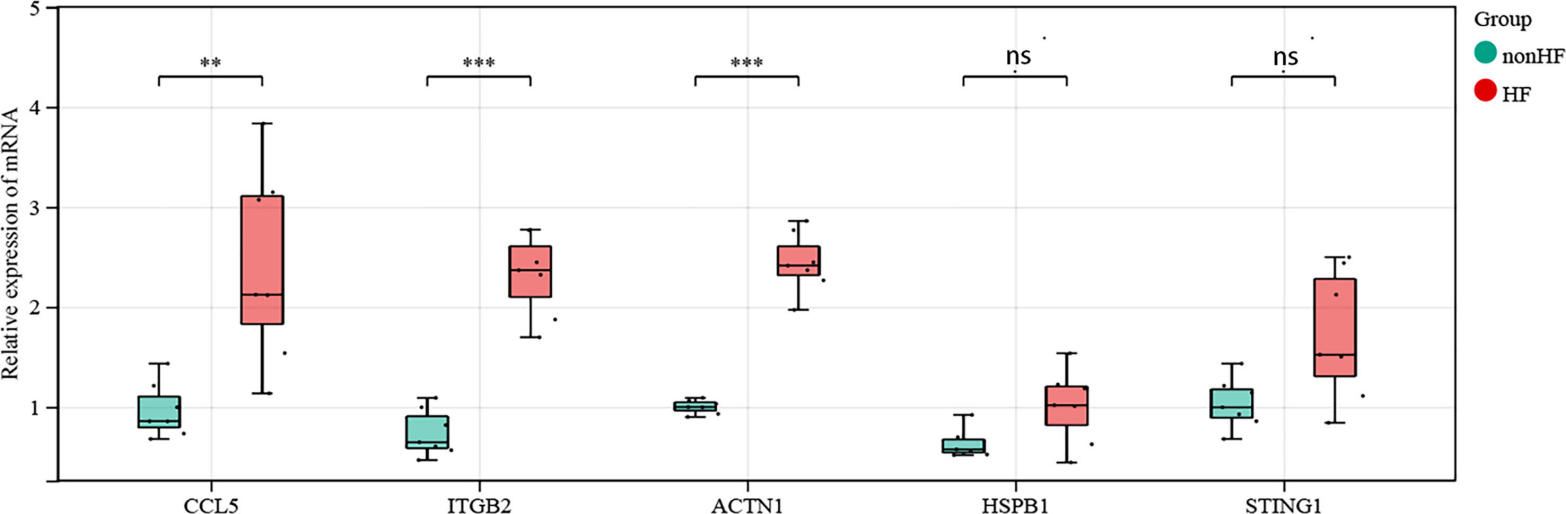
Validation of *CCL5, STING1, HSPB1, ACTN1* and *ITGB2* in human blood samples (non-HF: n = 7 biologically independent samples, HF: n = 7 biologically independent samples). Expression status of *CCL5, ACTN1* and *ITGB2* in the blood of post-MI patients. Expression status of *STING1* and *HSPB1* in the blood of post-MI patients. *Wilcoxon rank-sum test*, **p < 0.01, ***p < 0.001. ns, not statistically significant.

We compared the mRNA expression of *CCL5, STING, HSPB1, ACTN1*, and *ITGB2* in post-MI HF patients with that in post-MI non-HF patients using quantitative real-time polymerase chain reaction. *CCL5, ACTN1*, and *ITGB2* expression increased sharply in post-MI HF and non-HF blood samples, as predicted (P < 0.05) (**Figure 7**). When comparing patients with post-MI HF and patients without HF, both *STING* and *HSPB1* showed a significantly higher trend in the HF group than in the non-HF group, despite having p-values greater than 0.05 after the Wilcoxon rank-sum test (**Figure 7**).

## 4 Discussion

We reanalyzed a publicly available HF scRNA-seq dataset to explore the subtypes and characteristics of immune cells in the blood after MI to predict the risk of HF. We found that B cell subtype B_C4 is important for predicting whether HF will occur after MI. We used genetic markers associated with B_C4 to develop a signature, HF.Sig, consisting of 13 genes. Through gene enrichment analysis, we found that B_C4 was mainly associated with the activation of NK immune cells and the interaction between immune cells. The predictive ability of HF.Sig was systematically evaluated. A post-MI HF prediction model was developed using HF.Sig. By analyzing GSVA and differential gene expression, we further screened five genes (*STING1, HSPB1, CCL5, ACTN1*, and *ITGB2*) that were highly expressed in patients with post-MI HF. Through the verification of blood samples from clinical patients, the validity (significant differences in the identified genes between the HF and non-HF groups) of the genes we found was examined. We developed a predictive model for the prognosis of post-MI patients by combining scRNA-seq and bulk RNA-seq analyses. Given the ease of blood testing in the clinic, our HF signature is a powerful diagnostic tool for practical clinical applications to determine whether HF will occur after MI.

Our predictive model can predict the onset of post-MI HF and has important clinical implications for its prevention and treatment. For patients who are at risk of HF after MI, early diagnosis and treatment and early intervention in HF can greatly reduce mortality in patients who develop HF after MI. Strong evidence and practice guidelines suggest that interventional and pharmacological treatments primarily benefit high-risk patients (28, 29). Despite this guidance, identifying patients at high risk of cardiac ischemic events remains challenging (30, 31). Therefore, the prediction of post-infarction HF at the molecular level is particularly important.

The data in the scRNA dataset are from patients with post-MI HF compared to normal samples. The genes we get from the single cells may be related to MI and/or HF, so we use the MI data to verify the MI-related genes first, and then further identify the post-MI HF-related genes to establish a model gene for the diagnosis of post-MI HF. In summary, two-steps strategy guarantees the identification of post-MI HF-associated genes.

Immunoinflammatory pathways have a significant impact on the recovery of patients with HF and MI (32). B lymphocytes infiltrate the infarcted myocardium starting on day 1 and continue to do so until the healing period, according to earlier research using animal models of MI (day 7) (33). Reduced post-MI inflammation and improved functional cardiac outcomes in mice have been linked to B cell depletion using CD20-specific antibodies (34). The production of chemokines by B cells to draw in monocytes and create pathogenic antibodies has been demonstrated to aggravate the unfavorable remodeling of the myocardium; however, the exact processes by which B cells alter myocardial function are still poorly understood (34). In a model of chronic HF, transgenic mice unable to secrete immunoglobulin B cells displayed less cardiac remodeling and diminished cardiac function than normal mice (35, 36).

The activation of autophagy by *STING* has been demonstrated to occur through a mechanism involving *TBK1* activation and interferon induction (37), which serves as a steward for specific cytoplasmic protein breakdown and organelle recycling, both of which are essential for harmful cardiac remodeling (37). After MI, inhibition of the specific small molecule *STING1* may enhance the wound healing response and pathological remodeling, thereby lowering the occurrence of ischemic HF (38). Rech et al. demonstrated that inhibition of the specific small molecule *STING1* after MI may enhance the wound healing response and pathological remodeling, thereby lowering the occurrence of ischemic HF (38). *HSPB1* is a negative regulator of the cardiomyocyte inflammatory response and is essential for repairing injured heart tissue following myocardial infarction. The action of *HSPB1* is partially attributed to nuclear factor NF-κB-dependent regulation of leukocyte recruitment and subsequent inflammation (39). Another indicator of poor prognosis in patients with chronic HF is *HSPB1* upregulation (40). *CCL5* has been shown to be highly expressed in the peripheral circulation after MI, and blocking *CCL5* lowers serum levels of neutrophil and monocyte chemo-attractants during chronic myocardial ischemia while anti-*CCL5* monoclonal antibody therapy improves post-MI “clinical” outcomes, such as survival and cardiac function (41). *ITGB2* is a transmembrane adhesion and signaling receptor expressed only in leukocytes and extracellular vesicles. It promotes an inflammatory reaction and assists leukocytes in adhering to tissues and to move around (42, 43). Liu et al. demonstrated that suppressing the expression of *ITGB2* in macrophages in a mouse model of HF decreased the infiltration of myocardial immune inflammatory cells and cardiac hypertrophy (44). The inflammatory response after cardiac injury on the one hand provides protection against short-term adaptation of the heart. Nonetheless, this inflammatory response can also lead to left ventricular dysfunction and myocardial remodeling (45). Although Jia et al. identified multiple genes linked to myocardial contraction, cardiac hypertrophy, cardiac fibrosis, and myocardial damage, including *ACTN1*, the function of *ACTN1* in the pathogenesis of HF after MI has not yet been established (46). Several studies have used *ACTN1* as a marker of cardiogenesis (47, 48).

In conclusion, our study provides a valid predictive model for prognosis after MI using blood markers and explores the link between immune cells in the blood and outcomes. Given the ease with which blood specimens can be used in clinical practice, this study offers a powerful tool for the clinical use of MI. However, this study has some limitations. First, we only had one set of single-cell RNA-seq data and one set of bulk RNA-seq data related to HF after MI for analysis. Second, there were no clinical features other than disease information in the dataset used. We described the relationship between B cells in the blood and the presence of HF after MI but did not clarify the mechanism of their association. Future experiments with larger sample sizes and more rigorous designs are needed to explore the mechanisms between disease and cells to consolidate the findings of this study.

By analyzing both the single-cell and bulk datasets, we determined that B cells are reliable predictors of post-MI HF and identified their enrichment in several immune-related pathways. In this study, we established a diagnostic model for post-MI HF risk prediction consisting of 13 genes, of which, five genes were confirmed to be differentially expressed in the HF cohort using multiple methods. We proposed a highly effective method for predicting recovery after MI. We were only able to use one single-cell sequencing dataset and three infarct-related datasets. Therefore, this study has the limitation that there were fewer sequencing data that met our study’s requirements. However, by validating the polymerase chain reaction in clinical specimens, we identified multiple genes that are important to this model. In conclusion, we present a highly effective clinical diagnostic model that may offer a new perspective for the clinical diagnosis and treatment of MI.

## Data availability statement

The original contributions presented in the study are included in the article/**Supplementary Material**. Further inquiries can be directed to the corresponding authors.

## Ethics statement

The studies involving human participants were reviewed and approved by Cheeloo College of Medicine, Shandong University. The patients/participants provided their written informed consent to participate in this study. Blood samples for the validation experiment in which this study is based were obtained from patients admitted to the hospital with MI, including patients with and without HF. The study was approved by the Research Ethics Committee of Qilu Hospital (reference number: KYLL-2021[KS]-393), in compliance with the Declaration of Helsinki and the Code of Ethics of the World Medical Association. Written informed consent for participation in the study was obtained from all patients.

## Author contributions

Conceptualization: XG, HS, and XZ. Methodology: XZ. Investigation: XZ and XK. Project administration: HS and XZ. Writing of the original draft: XK, HS, and XZ. All authors conceived and designed the study. All authors contributed to the article and approved the submitted version.
