# Supplementary material for: Risk prediction model construction for post myocardial infarction heart failure by blood immune B cells
Source: Front Immunol. 2023 May 23;14:1163350. doi: 10.3389/fimmu.2023.1163350 (PMC10242647; doi:10.3389/fimmu.2023.1163350)
Supplement: Supplementary file 1 [file DataSheet_1.docx]

**Risk prediction model construction for post myocardial infarction heart failure by immune B cells in blood**

**HouRong Sun1,2†, XiangJin Kong1,2†, LingWei Meng1,2, Jie Hao 4, Yue Xi 5, KaiMing Wei1,2, GuanNan Li1,2 and Xin Lv1,2, Xin Zou3* and XingHua Gu1,2***

**Supplementary materials**


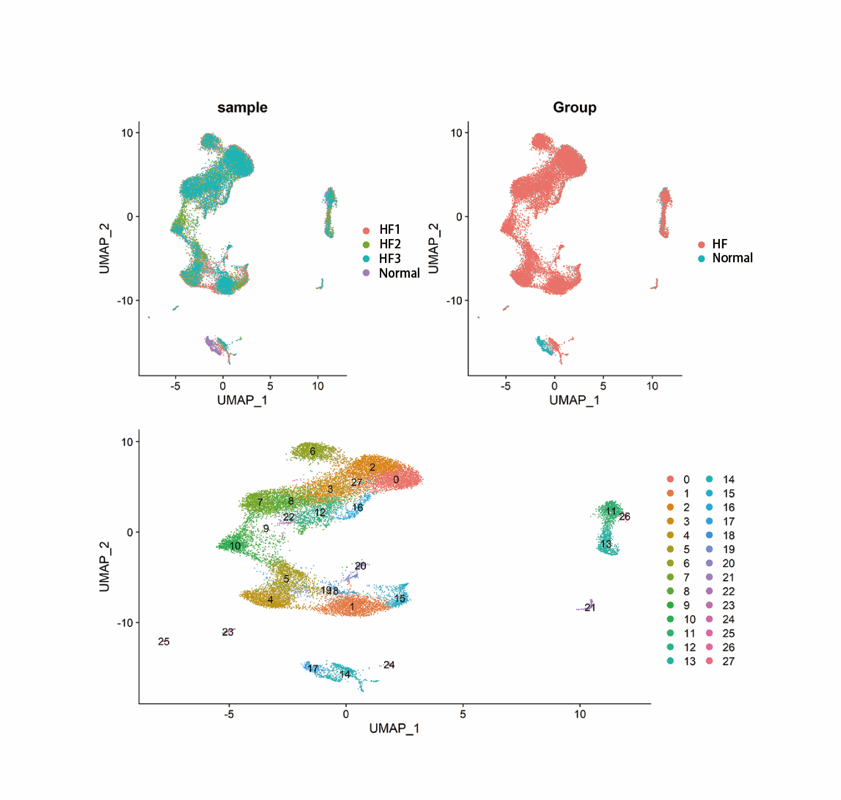


**Supplementary Figure.1** UMAP scatter plot of single cell data. UMAP scatter plot of single cell data according to total cell sub-clusters between myocardial infarction resulting in heart failure and normal subjects. UMAP scatter plot of cell distribution after completion of annotation of cell subclusters.


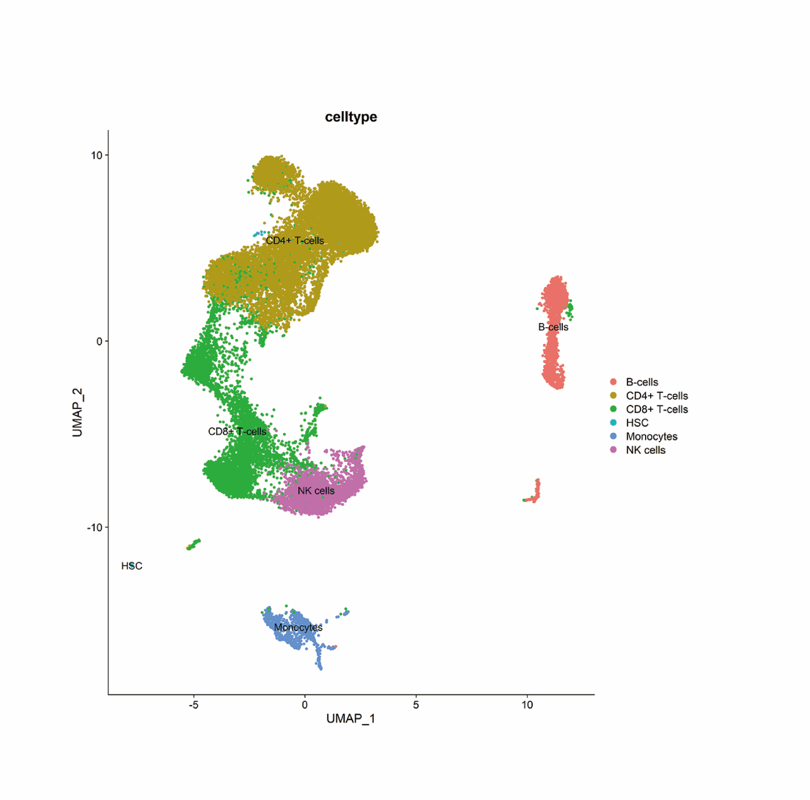


**Supplementary Figure.2** The annotation of cell subsets was visualized using umap diagrams, with a total of 6 types of cells annotated


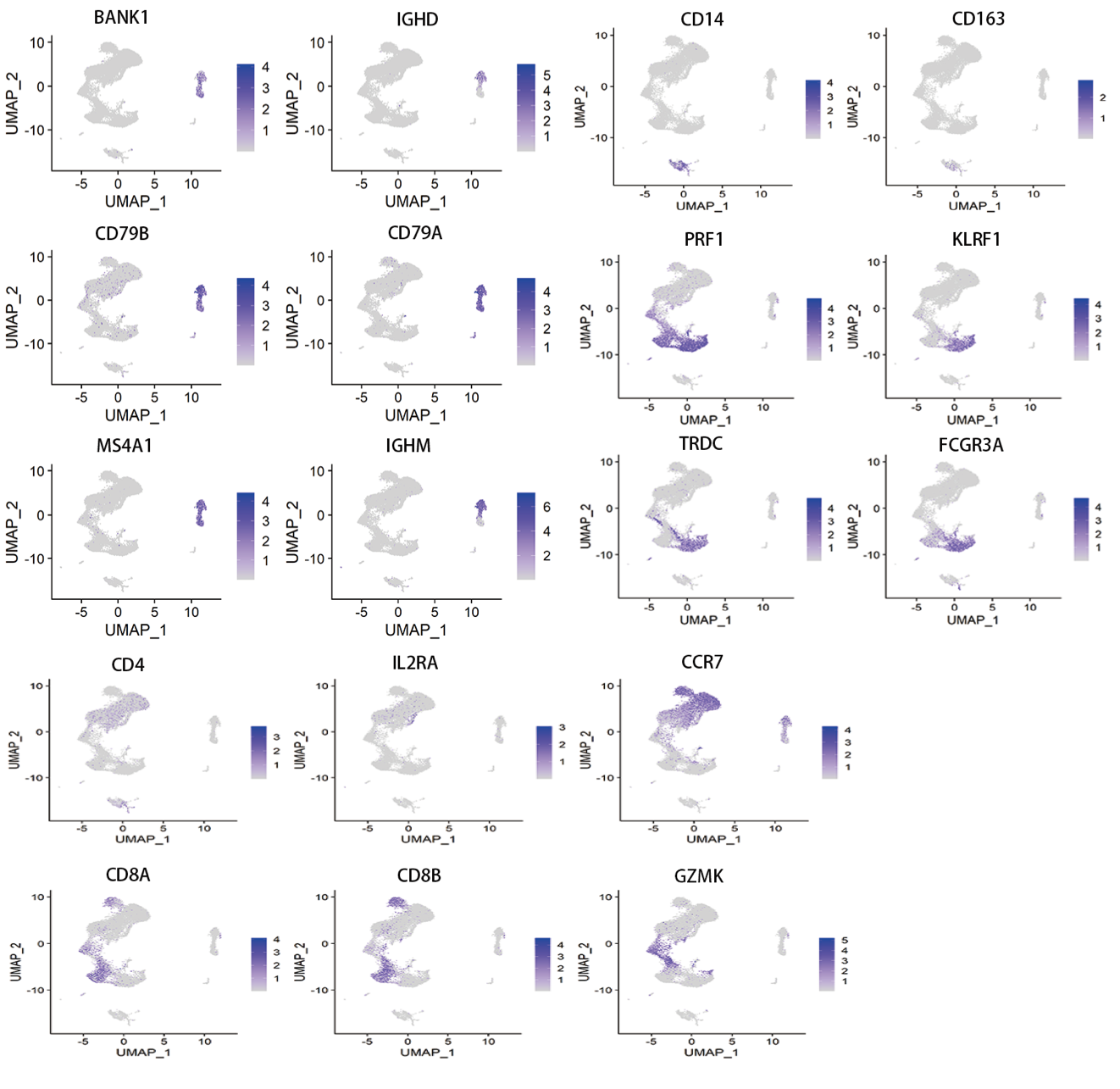


**Supplementary Figure.3** Scatter plot of the distribution of specific markers for each cell type in the UMAP. Specific markers for CD8T cells, CD4T cells, B cell, NK cells and monocytes.


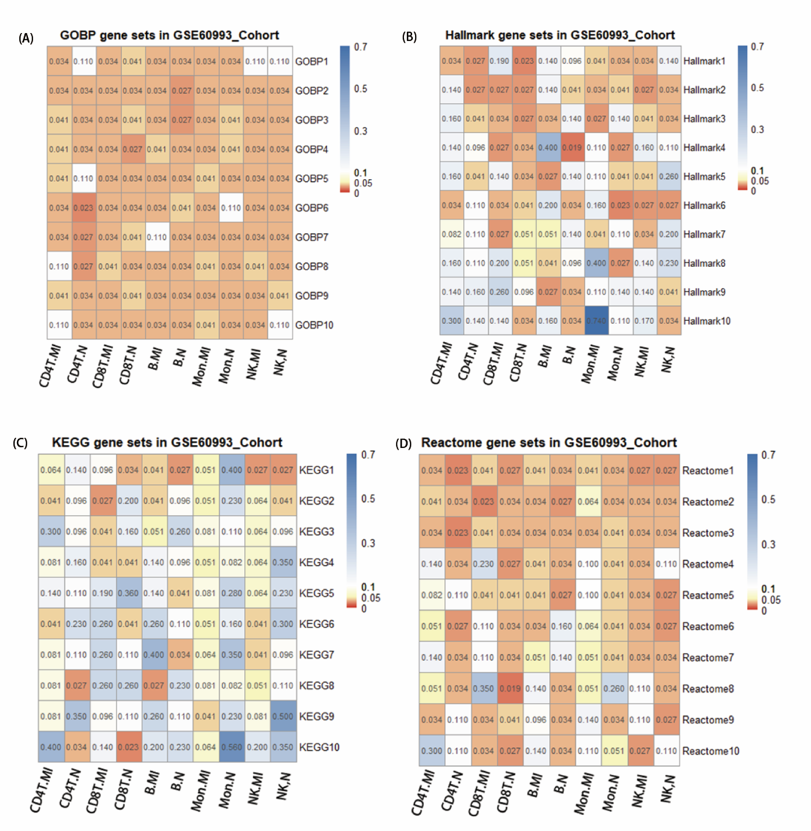


**Supplementary Figure.4** The performances of 10 DE gene list-enriched gene sets for predicting MI and identification of MI-related cell types. The scRNA-seq datasets and bulk RNA-seq (GSE61144) cohort were analyzed. (A)-(D) p values of receiver operating characteristic (ROC) curves for predicting MI of 10 DE gene-list enriched (A) GOBP, (B) Hallmark, (C) KEGG and (D) Reactome gene sets. MI for myocardial infarction and N for normal control in the bulk data.


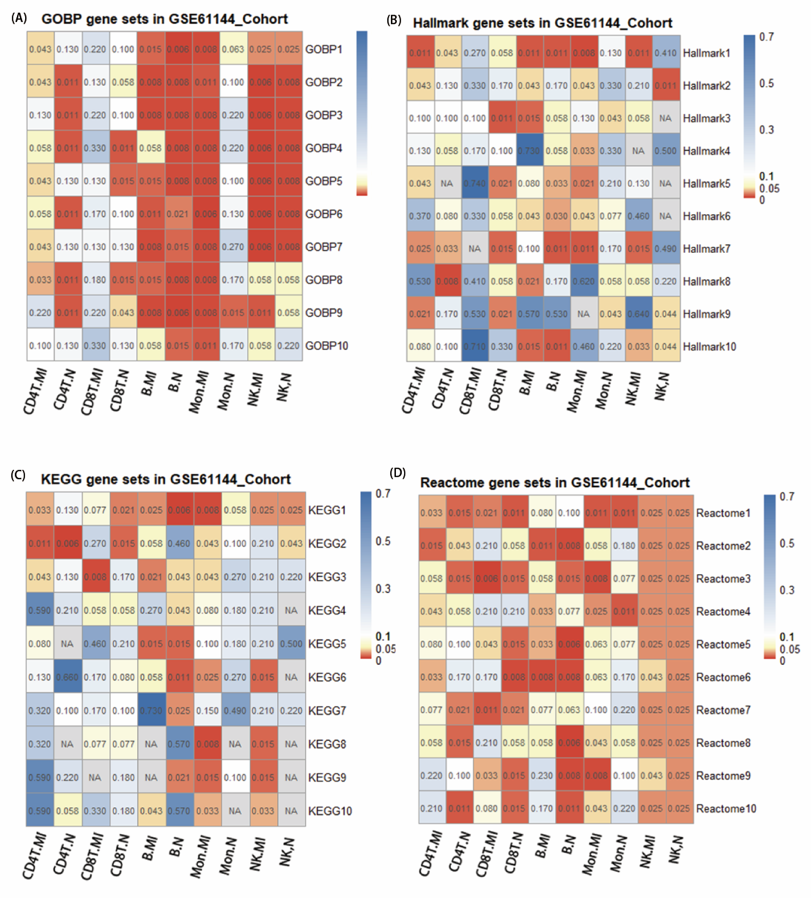


**Supplementary Figure.5** The performances of 10 DE gene list-enriched gene sets for predicting MI and identification of MI-related cell types. The scRNA-seq datasets and bulk RNA-seq (GSE60993) cohort were analyzed. (A)-(D) p values of receiver operating characteristic (ROC) curves for predicting MI of 10 DE gene-list enriched (A) GOBP, (B) Hallmark, (C) KEGG and (D) Reactome gene sets. MI for myocardial infarction and N for normal control in the bulk data.


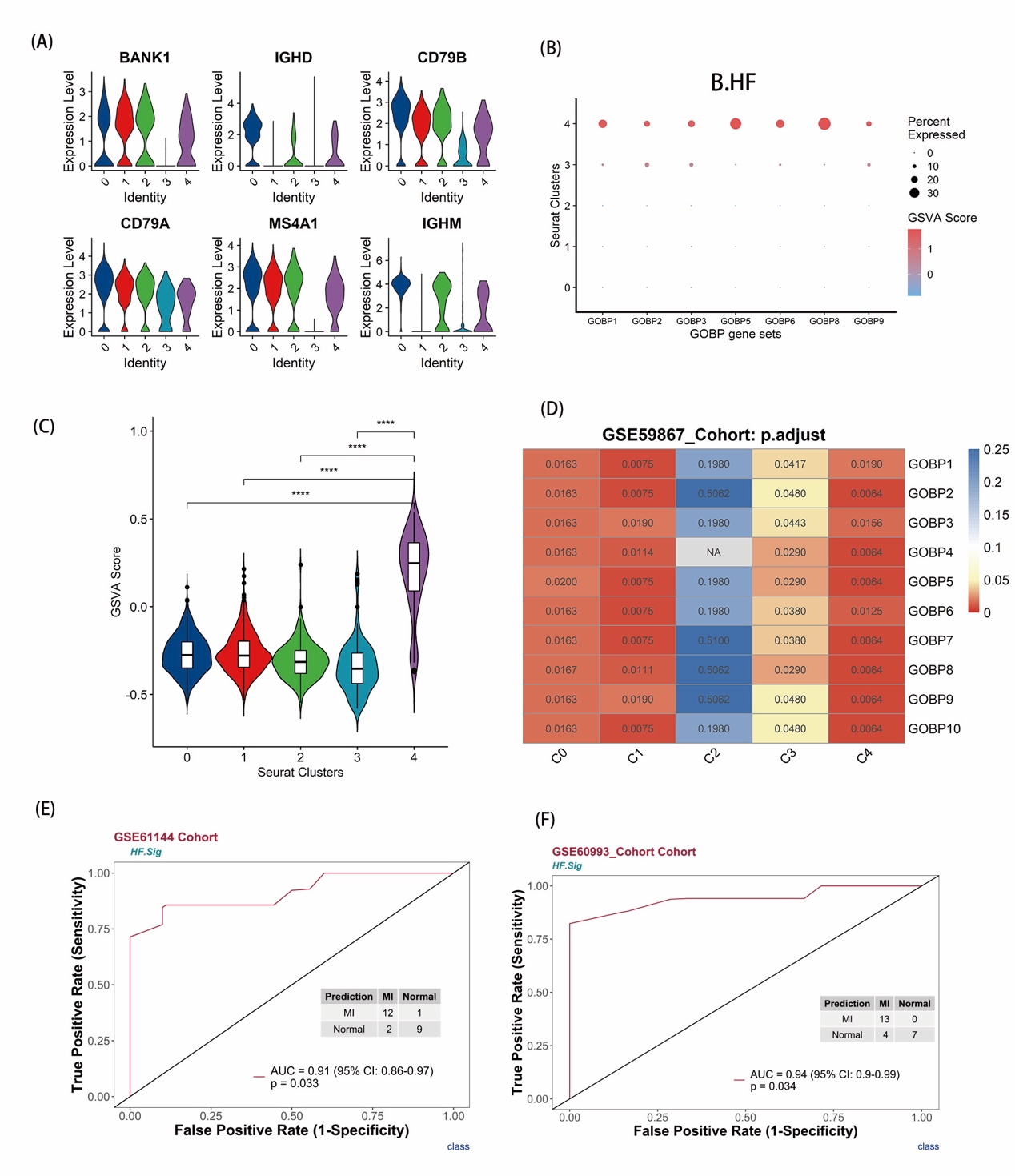


**Supplementary Figure.6** GSVA analysis of B cells and examination of HF.Sig (A) Violin plot of the expression levels of classical B cells marker genes in subclusters. (B) Dot plot of the expression levels of 7 gene sets enriched in B cells. MI with ROC adjusted p values < 0.05 in B cells via GSVA analysis. Related to Figure.2. (C) Violin of HF.Sig GSVA scores showed that it can specifically characterize B cells of C4. Center line, median. Box limits, upper and lower quartiles. Whiskers, 1.5 interquartile range. Points beyond whiskers, outliers. A two-sided Wilcoxon test was used to determine significance. ****P < 0.0001. (D) The performances of 5 DE gene list-enriched gene sets for predicting post-MI HF in MI-related cell types. Compared to other subclusters of B cells, the C4 cluster was the most effective in predicting post-MI HF. (E) and (F) was also shown that HF.Sig also have excellent performance in predicting MI.


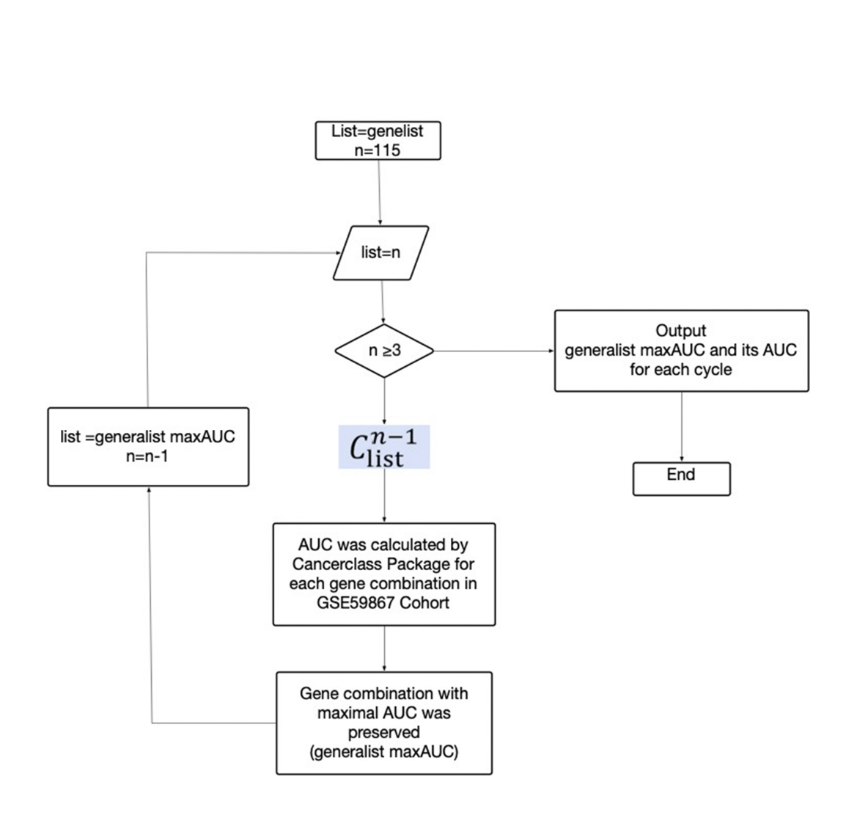


**Supplementary Figure.7** Circulation algorithm flow for Sig in post-MI HF.


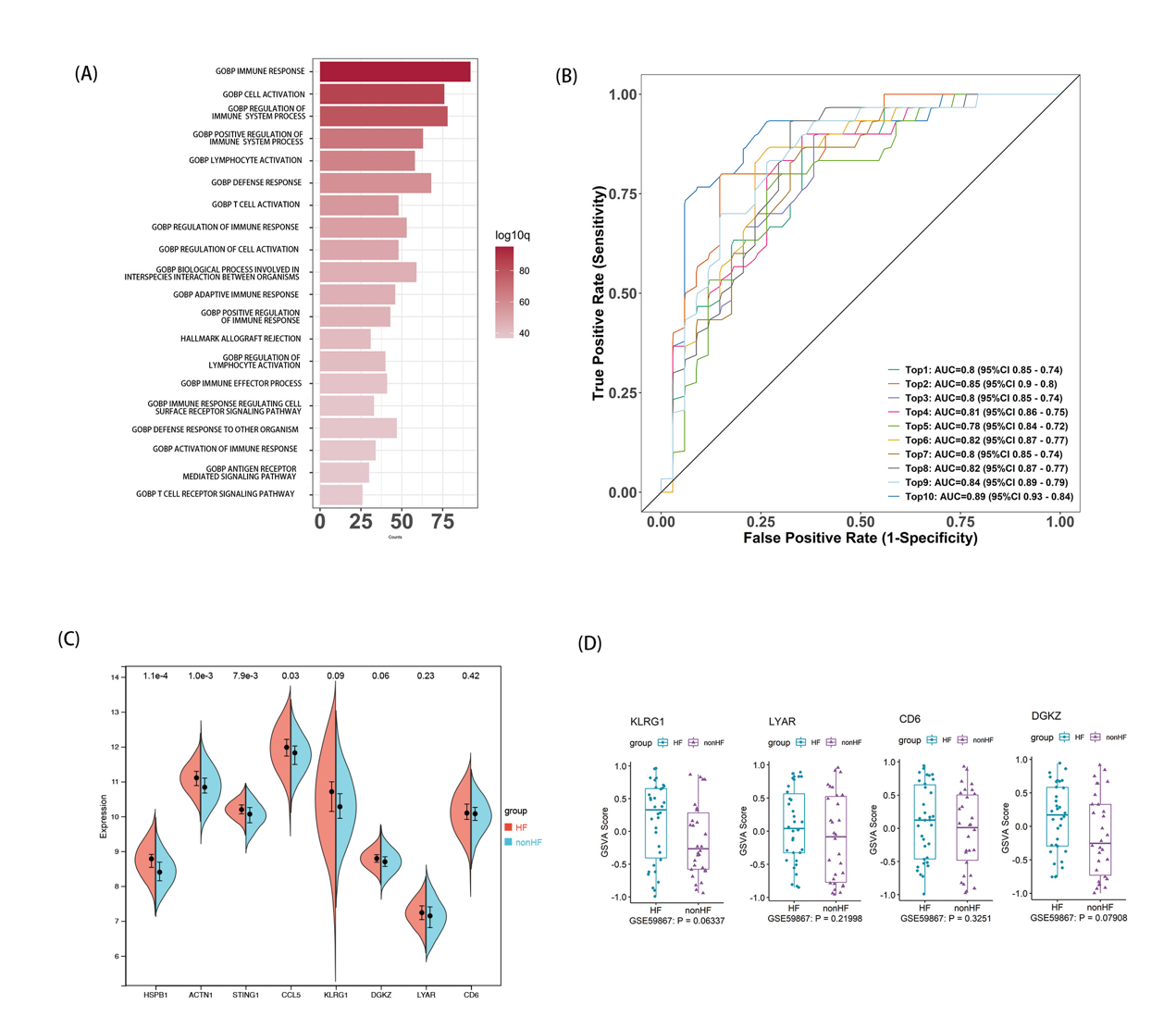


**Supplementary Figure.8** Analysis of gene enrichment of B cell associated marker and 8 associated genes (A) Top 20 pathways in gene enrichment for 115 genes in B_C4 marker. (B) The first ten pathways have excellent performance capabilities. (C) Optimized expression of 8 of the 13 genes in HF.Sig in GSE59867. (D) GSVA showed no significance for 4 genes in GSE59867.


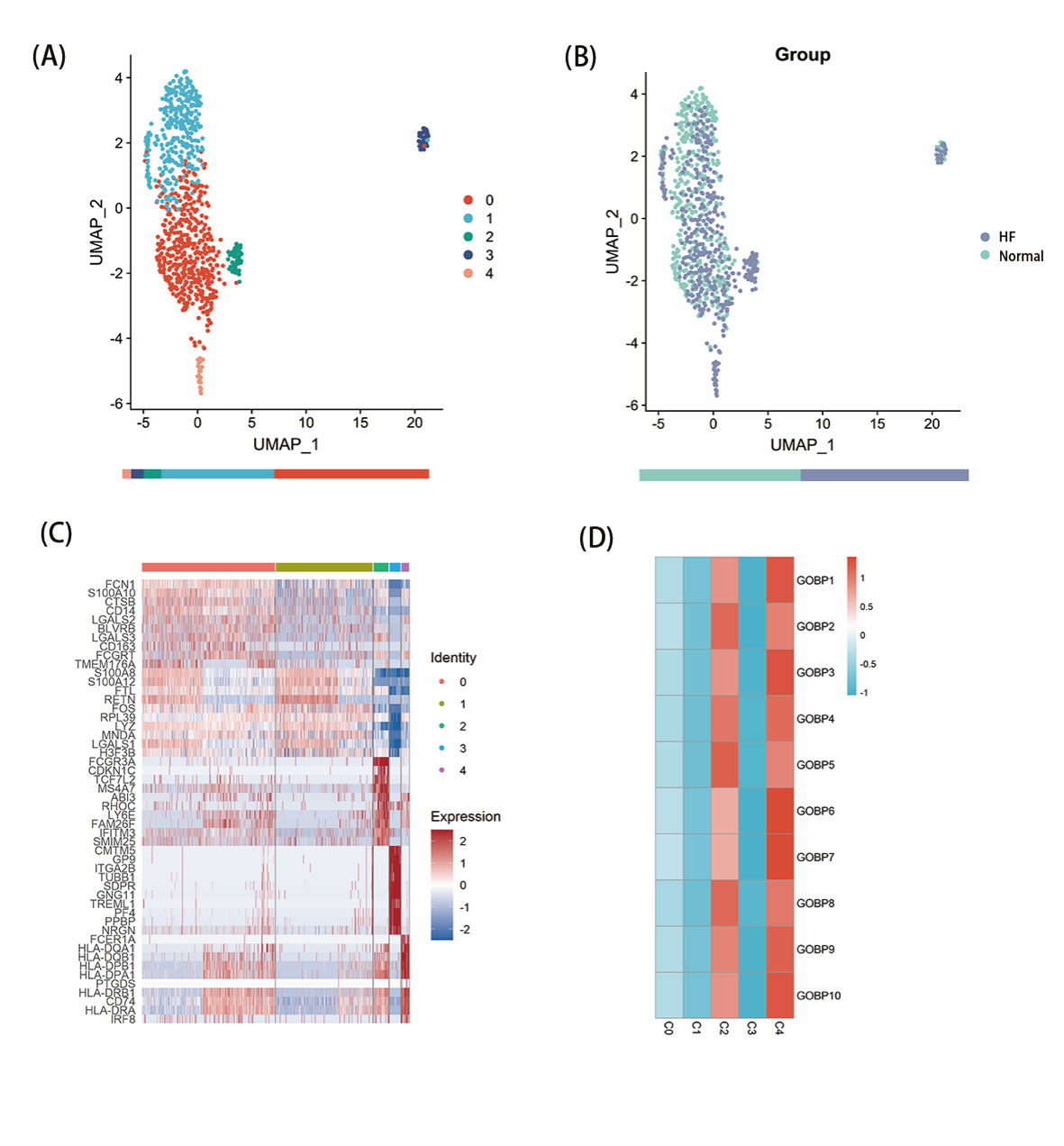


**Supplementary Figure.9** Correlation analysis of monocytes (A) UMAP plot of monocytes cells in the dataset of GSE145154, with the sample divided into five subgroups. (B) two subgroups were divided according to disease status. (C) Heatmap of scale-normalized expression of the top 10 specific marker genes for the monocytes-cell subclusters in GSE145154, identified by a two-sided Wilcoxon rank sum test with FDR correction (q < 0.05). (D) Identification of MI prediction associated monocytes subclusters by gene set variance analysis (GSVA) to locate valid (ROC p.adjust < 0.05) predicted gene set expression.


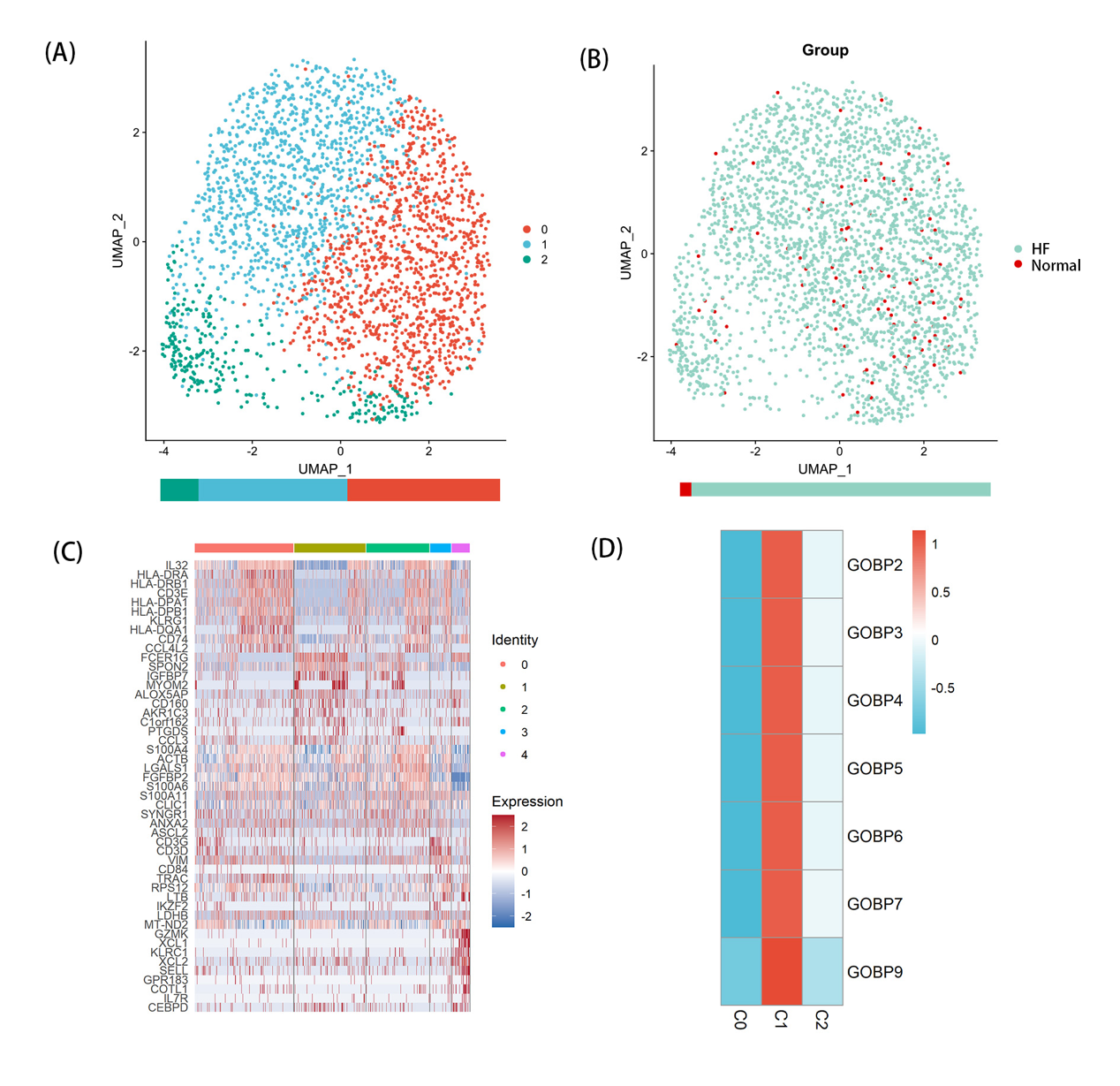


**Supplementary Figure.10** Correlation analysis of NK cells (A) UMAP plot of NK cells in the dataset of GSE145154, with the sample divided into five subcluster. (B) Two subcluster were divided according to disease status. (C) Heatmap of scale-normalized expression of the top 10 specific marker genes for the NK-cell subclusters in GSE145154, identified by a two-sided Wilcoxon rank sum test with FDR correction (q < 0.05). (D) Identification of MI prediction-associated NK cell subclusters by gene set variance analysis (GSVA) to locate valid (ROC p.adjust < 0.05) predicted gene set expression.


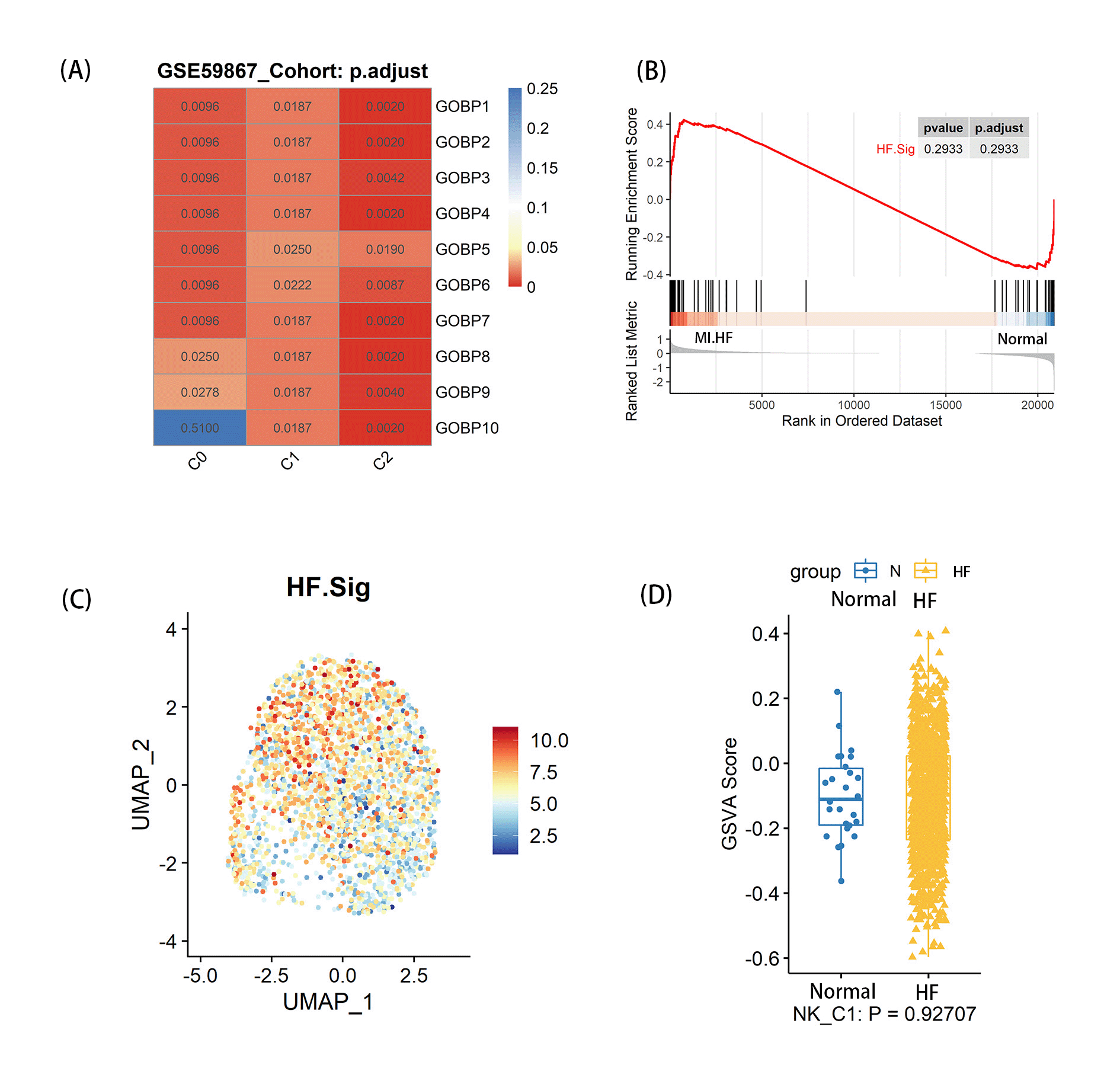


**Supplementary Figure.11** (A) The performances of 5 DE gene list-enriched gene sets for predicting post-MI heart failure in MI-related cell types. C1 cell subclusters have good predictive performance. (B) GSEA analysis revealed that HF.Sig of NK cells could not be meaningfully enriched in NK_C1 and enriched in HF and Normal, p = 0.2933, p value adjusted by Benjamini-Hochberg method for FDR. (C) Feature plot of the HF.Sig GSVA score shows that it cannot specifically describe the NK.Sig. (D) Boxplot found and verified that the MI score in the NK HF.Sig GSVA score is not significantly different from the Normal score.
